# Methods to maximise recovery of environmental DNA from water samples

**DOI:** 10.1371/journal.pone.0179251

**Published:** 2017-06-12

**Authors:** Rheyda Hinlo, Dianne Gleeson, Mark Lintermans, Elise Furlan

**Affiliations:** Institute for Applied Ecology, University of Canberra, Bruce, ACT, Australia; University of Hyogo, JAPAN

## Abstract

The environmental DNA (eDNA) method is a detection technique that is rapidly gaining credibility as a sensitive tool useful in the surveillance and monitoring of invasive and threatened species. Because eDNA analysis often deals with small quantities of short and degraded DNA fragments, methods that maximize eDNA recovery are required to increase detectability. In this study, we performed experiments at different stages of the eDNA analysis to show which combinations of methods give the best recovery rate for eDNA. Using Oriental weatherloach (*Misgurnus anguillicaudatus*) as a study species, we show that various combinations of DNA capture, preservation and extraction methods can significantly affect DNA yield. Filtration using cellulose nitrate filter paper preserved in ethanol or stored in a -20°C freezer and extracted with the Qiagen DNeasy kit outperformed other combinations in terms of cost and efficiency of DNA recovery. Our results support the recommendation to filter water samples within 24hours but if this is not possible, our results suggest that refrigeration may be a better option than freezing for short-term storage (i.e., 3–5 days). This information is useful in designing eDNA detection of low-density invasive or threatened species, where small variations in DNA recovery can signify the difference between detection success or failure.

## Introduction

The past decade has seen a remarkable growth and interest in the use of the environmental DNA (eDNA) method as a tool for targeted species detection and biodiversity assessments. While earlier research focused on the isolation of microbial DNA from environmental samples [1, 2], subsequent studies directed at isolating plant and animal DNA from such samples has opened up diverse fields of application of the method including surveillance of rare, threatened, or invasive species [3–5] and assessment of past and present biodiversity [6–8]. Many eDNA studies have been in aquatic ecosystems, and have proven effective in the detection of aquatic/semi-aquatic vertebrates and invertebrates [9–14]. Although the application of this non-invasive genetic technique is increasingly expected to influence environmental management [15], basic studies are encouraged to further explore its possibilities and limitations [16].

The eDNA method relies on the detection of DNA in environmental samples such as water, soil or air to infer species presence [17]. Its analysis involves a series of steps, which includes eDNA capture, preservation, extraction, amplification and sequencing to ensure match to target species. Efficiency at each step is expected to affect the recovery of DNA and subsequently, its detection. Indeed, previous research found that eDNA recovery varied depending on the protocols or combinations of protocols used [18–21]. Researchers often choose methods based on cost, ease of sampling, availability of materials and equipment or personal preferences [18, 21]. Different combinations of methods are likely to vary in efficiency but because eDNA analysis often relies on detecting small quantities of highly degraded DNA, methods that maximize eDNA recovery (thus, increasing detection) in a cost-efficient manner are ideal.

A crucial first step in the eDNA workflow is DNA capture. Filtration and precipitation are the two most commonly used methods to capture eDNA from aquatic environments. Filtration requires passage of water samples through a filter to trap the DNA whereas the precipitation method uses ethanol to precipitate nucleic acids in the water sample [3, 22]. Comparisons of these two methods found that filtration recovered more eDNA from water samples [18, 19, 21]. The precipitation method limits the amount of water volume that can be processed, and thus filtration has been suggested to be more advantageous when dealing with larger bodies of water [23]. A few studies have also investigated how different DNA capture and extraction combinations affect eDNA yield [18, 19, 21]. These studies however did not look at preservation/storage method along with capture and extraction methods which may also affect DNA recovery.

After filtration, DNA has to be preserved prior to DNA extraction. Cold storage of filter papers is commonly employed [3, 10], although this may be impractical in some field applications. Ambient temperature storage has been successfully employed using ethanol [24–26] and Longmire’s solution [20, 27]. Ambient temperature storage is useful when maintaining a cold chain in field conditions is difficult. Of these two alternatives, ethanol is likely to be the preferred alternative as it is widely available, inexpensive and can be used straightaway with no preparations needed. Studies comparing eDNA recovery rates from filters stored under cold storage and those stored in ethanol, however, are limited [27, 28].

In studies using filtration as a capture method, water samples are taken from the field, brought to the laboratory in ice chests, and filtered within 24 hours or are frozen before processing [14, 29]. There may be instances, however, when water samples cannot be processed within 24h of collection. In this case, it is important to know how long samples can be stored without significantly affecting detection or DNA concentration. Previous studies have shown that eDNA degrades exponentially with time [8, 30]. Storing water samples in freezers seem to be the preferred option but access to freezers are not always available and the freeze-thaw cycle has been found to affect DNA detection [31]. The current published study measured the eDNA concentration after one freeze-thaw cycle only [31] but no other study has investigated the effect of several freeze-thaw cycles on eDNA over a period of time. The effect of sample processing (time and method of storage before filtration) on eDNA recovery has previously been investigated [30], but using only limited storage methods (ambient temperature, frozen) and within a relatively short time frame (up to four hours) [30, 31]. Our study aims to address these gaps by investigating the effect of three storage methods (room temperature, refrigerated and frozen) on eDNA across a longer period of time (28 days).

The type and pore size of filter papers used during filtration in eDNA studies also vary. These are important considerations in eDNA analysis as filter material and pore size directly influence flow rate and particle retention. Many eDNA studies use pore sizes ranging from 0.45μm– 3 μm [9, 10, 32–34]. For highly turbid water however, such as in the tropics, even 3 μm filter papers are easily blocked, necessitating the use of larger pore size filters or pre-filtration to significantly minimize filtration time [35]. Glass fibre (GF), cellulose nitrate (CN), polycarbonate (PC), nylon, polyethersulfone (PES) and cellulose acetate (CA) filters have been used in eDNA studies [36]. Because filter papers are made from different materials, we expect that DNA would bind to each filter paper type differently. Indeed, Liang and Keeley [37] found that DNA had different binding affinities to different filter papers. Results from previous studies suggest an interaction effect of filter paper type and extraction method on DNA yield [19, 20]. These studies however used a limited number of filter papers and with different pore sizes, making direct comparison of DNA yields difficult. One of the aims of this study is to compare different types of filter paper with similar pore sizes so that direct comparisons on eDNA yield can be made.

It is apparent that the combinations of capture, storage and DNA extraction methods influence the final detection/quantity of eDNA. In this study, we performed experiments to compare eDNA recovery at different stages of the eDNA analysis in order to determine which methods are most cost-efficient. We used a rapidly-expanding invasive species in Australia, the Oriental weatherloach (*Misgurnus anguillicaudatus*) as a study species. Although there are many methods available, we chose to compare methods and materials that are widely available and will not require specialized equipment or unnecessarily increase processing costs. Specifically, we investigate DNA recovery obtained from: different combinations of capture (filtration and precipitation/centrifugation method), preservation (freezing vs ethanol) and DNA extraction (DNeasy and PowerWater) methods; 2) different combinations of commercial DNA extraction kit and filter paper type, and; 3) different storage methods and time.

## Methods

We performed experiments in controlled conditions in aquaria. Oriental weatherloach was used as a study species because its small size and hardiness makes it amenable to aquarium manipulation and because of its immediate management requirements in Australia. The Oriental weatherloach is a pest fish that is expanding its range in the country [38]. Sensitive detection using eDNA will assist management efforts, particularly during the early stages of invasion.

The Oriental weatherloach used for the experiments were obtained from Ginnindera Creek, Australian Capital Territory (ACT), through the ACT government scientific license LT2013661 and LT2014755 (granted to Elise Furlan). The fish were kept in a 60L plastic holding tank (∼15 individuals per container) at the Animal House facility at the University of Canberra (UC). The tank was continuously filtered and aerated and the fish were fed a commercial diet (bloodworms) once a day. The temperature was held at 20°C under a 12 h∶12 h light-dark cycle. This study was performed in compliance with the Australian code for the care and use of animals for scientific purposes (8^th^ edition 2013). The primary author was granted the authorization to conduct experiments using animals by the UC Animal Ethics Committee (Auth 14–09).

### Experiment 1: Comparison of different DNA capture, preservation and extraction combinations

**Fig 1:**
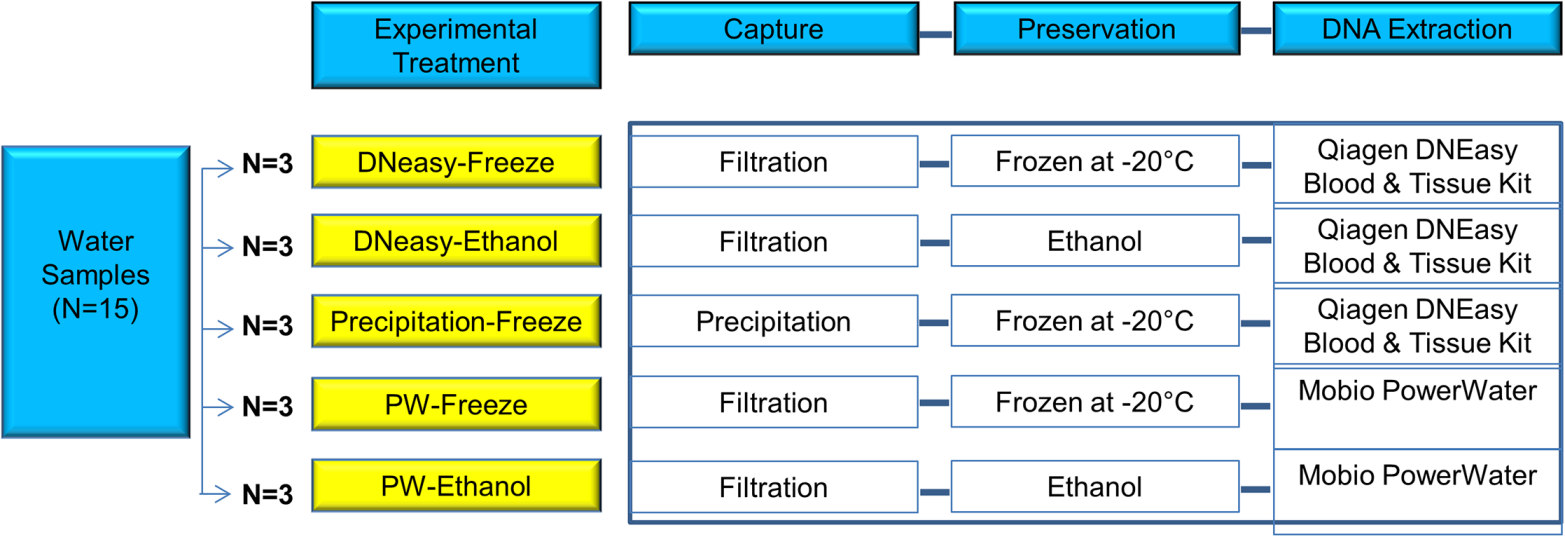
Experimental design used to compare five different combinations of capture, preservation and DNA extraction methods. N is the number of technical replicates and each replicate represents one 250 ml water sample. DNeasy and PowerWater (PW) refer to the two DNA extraction kits used in the experiment.

We compared the DNA yield of five different combinations of capture, preservation and DNA extraction methods (Fig 1). For DNA extraction, we included two commonly used commercial DNA kits. Although DNA kits are more expensive, they are advantageous in that they provide a standardised set of reagents and are easier and safer to handle compared to Phenol-Chloroform-Isoamyl alcohol (PCI) extraction reagents. Three 2-L water samples were taken from an aerated 40-L aquarium tank containing five adult Oriental weatherloach (*Misgurnus anguillicaudatus*). The water samples were inverted several times to ensure mixing and even distribution of contents before taking five 250mL aliquots. This created a total of 15 x 250mL water samples that were then randomly allocated across five treatment groups, permitting three technical replicates per treatment. For treatments requiring filtration, the 250ml aliquots were filtered through 47mm, 0.8um cellulose nitrate filter paper using a filter funnel manifold (Pall Australia Pty Ltd) and a peristaltic pump (Geopump®, Geotech, Colorado, USA). The filter papers were then either preserved in 100% ethanol at room temperature, or placed inside a -20°C freezer (depending on the treatment). For the treatment requiring precipitation, a 15 ml aliquot was obtained from each 250mL water sample and treated according to the method of Ficetola, Miaud [22]. Briefly, 1.5 ml of 3M sodium acetate and 33 ml absolute ethanol were added to the 15-ml aliquot and stored in a -20°C freezer prior to extraction. Four days after sample collection, DNA extraction was conducted on samples using either Qiagen’s DNeasy Blood and Tissue kit (Qiagen GmbH, Hilden, Germany) or PowerWater DNA Isolation Kit (Mo Bio Laboratories, Carlsbad, CA). DNA extraction with the DNeasy kit followed Renshaw et al.’s (2015) modification except we eluted the DNA in 200 μl buffer AE (Qiagen). Extraction with the PowerWater kit proceeded according to the kit’s protocol, including elution of the DNA in 100 μl of PW 6 (MoBio).

### Experiment 2A: Comparison of different combinations of filter paper and extraction method using water samples from aquaria with tap water

The eDNA yield from five types of filter papers of similar pore size (0.8um) and extracted with two types of DNA extraction kits were compared. The filter paper types investigated include cellulose nitrate (CN), mixed cellulose ester (MCE), polyethersulfone (PES), polycarbonate track-etched (PCTE), and glass fibre (GF). The filter papers were extracted with either Qiagen’s DNeasy Blood and Tissue kit (Qiagen, Hilden, Germany) or PowerWater DNA Isolation Kit (Mo Bio Laboratories, Carlsbad, CA). DNA extraction with the DNeasy and the PowerWater kits proceeded in the same manner as Experiment 1.

**Fig 2:**
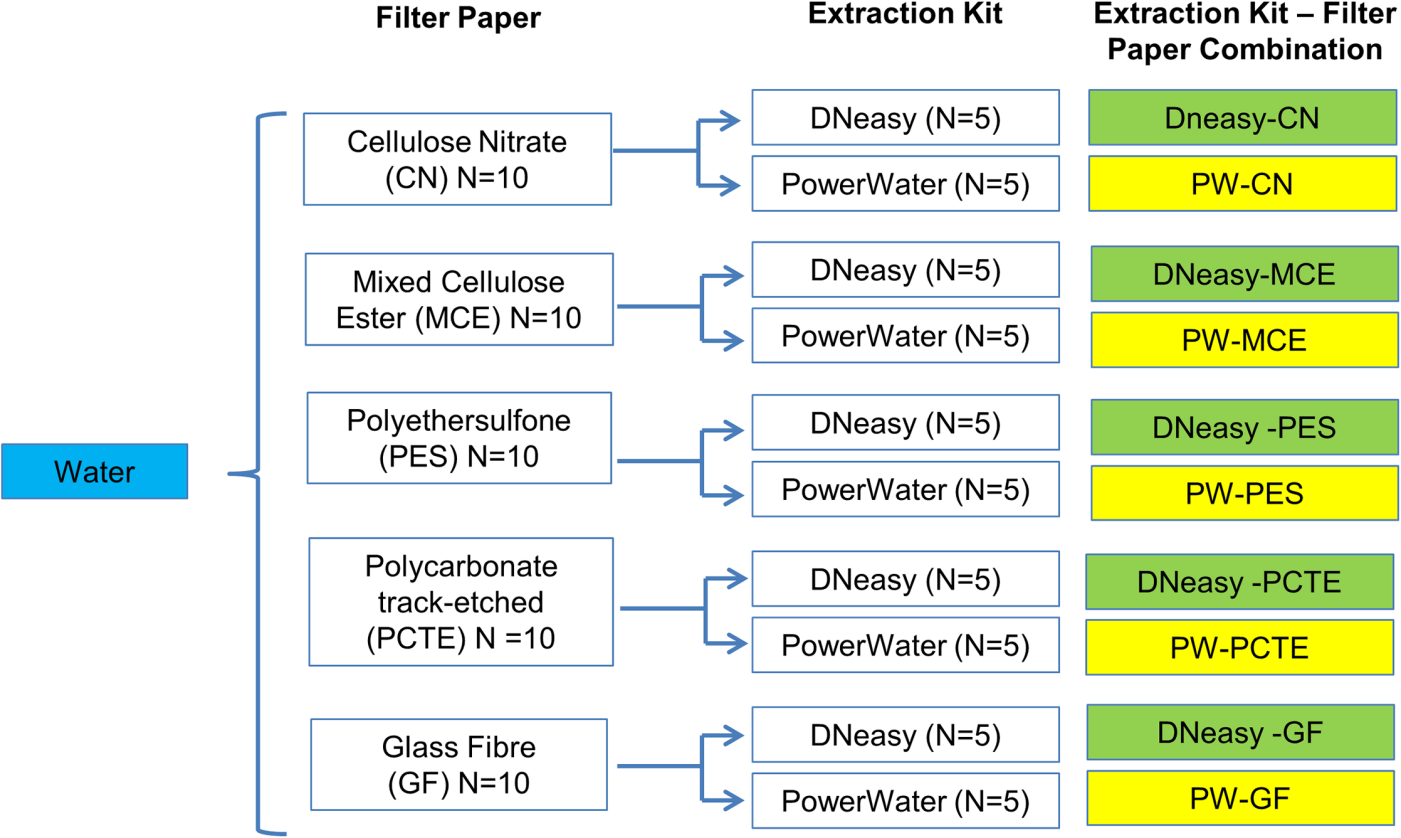
Experimental design for Experiment 2A to investigate which DNA extraction kit-filter paper combination would give the best DNA yield. *N* refers to number of technical replicates. CN, MCE, PES, PCTE and GF refer to the types of filter papers whereas DNeasy and PowerWater (PW) refer to the two DNA extraction kits used in the experiment.

We took five 2-L surface water samples from an aerated 40-L aquarium tank containing five adult Oriental weatherloach. From each of these water samples, ten 200-ml aliquots were taken and randomly allocated to a filter paper-DNA kit combination. This gave five replicates for each combination. The aliquots were filtered through 47mm, 0.8 μm pore size filter papers (Fig 2).

### Experiment 2B: Comparison of the best performing extraction kit-filter paper combinations using stream water

**Fig 3:**
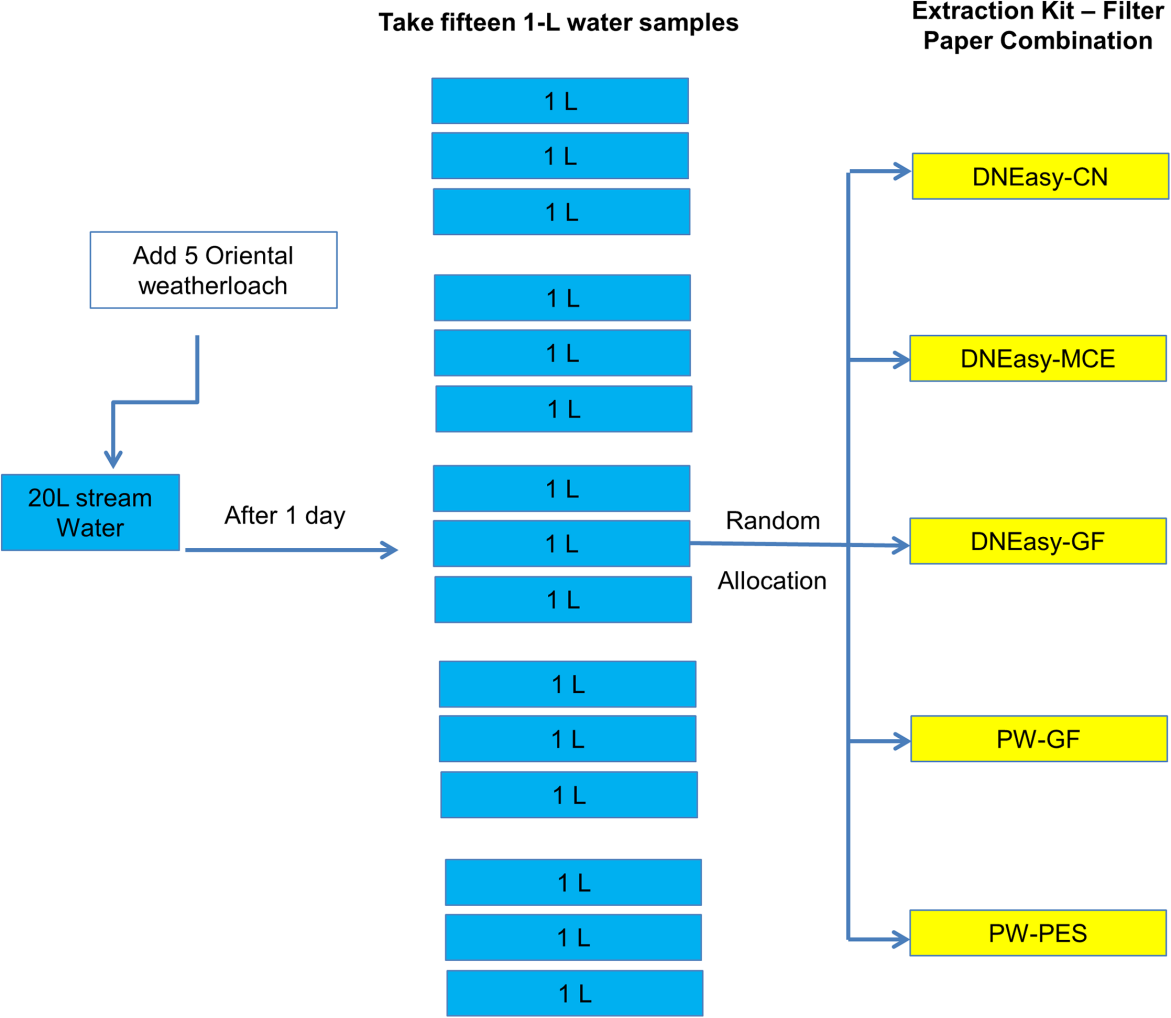
Experimental design for Experiment 2B investigating DNA yields in five different DNA extraction kit-filter paper combination from stream water. *N* refers to number of technical replicates. CN, MCE, PES, PCTE and GF refer to the types of filter papers whereas DNeasy and PowerWater (PW) refer to the two DNA extraction kits used in the experiment.

We took the top three performing DNA extraction kit-filter paper combinations from Experiment 2A and compared them using stream water to validate our findings. Because stream water would potentially contain more particulate matter compared to tap water and sample filtration time can differ depending on the filter paper types, we also recorded the time it took to filter 500ml of stream water on each filter paper type. We used 47mm, 1.2 μm pore size filter papers so that we can include 1.2 μm glass fibre (GF) filter papers since the thickness of the 0.8 μm GF caused samples to leak from the filter funnels during filtration. Briefly, we took 20 L of water from a natural stream (Gibraltar Creek, ACT, Australia). We then transferred this water to a 50L plastic tank and placed five adult Oriental weatherloach therein. After one day, we took 15 1-L samples from the tank and assigned the samples randomly to an extraction kit-filter paper combination (Fig 3). DNA extraction proceeded in the same manner as Experiment 1.

### Experiment 3A: Effect of storage method and time on eDNA concentration of water samples from aquaria with tap water

**Fig 4:**
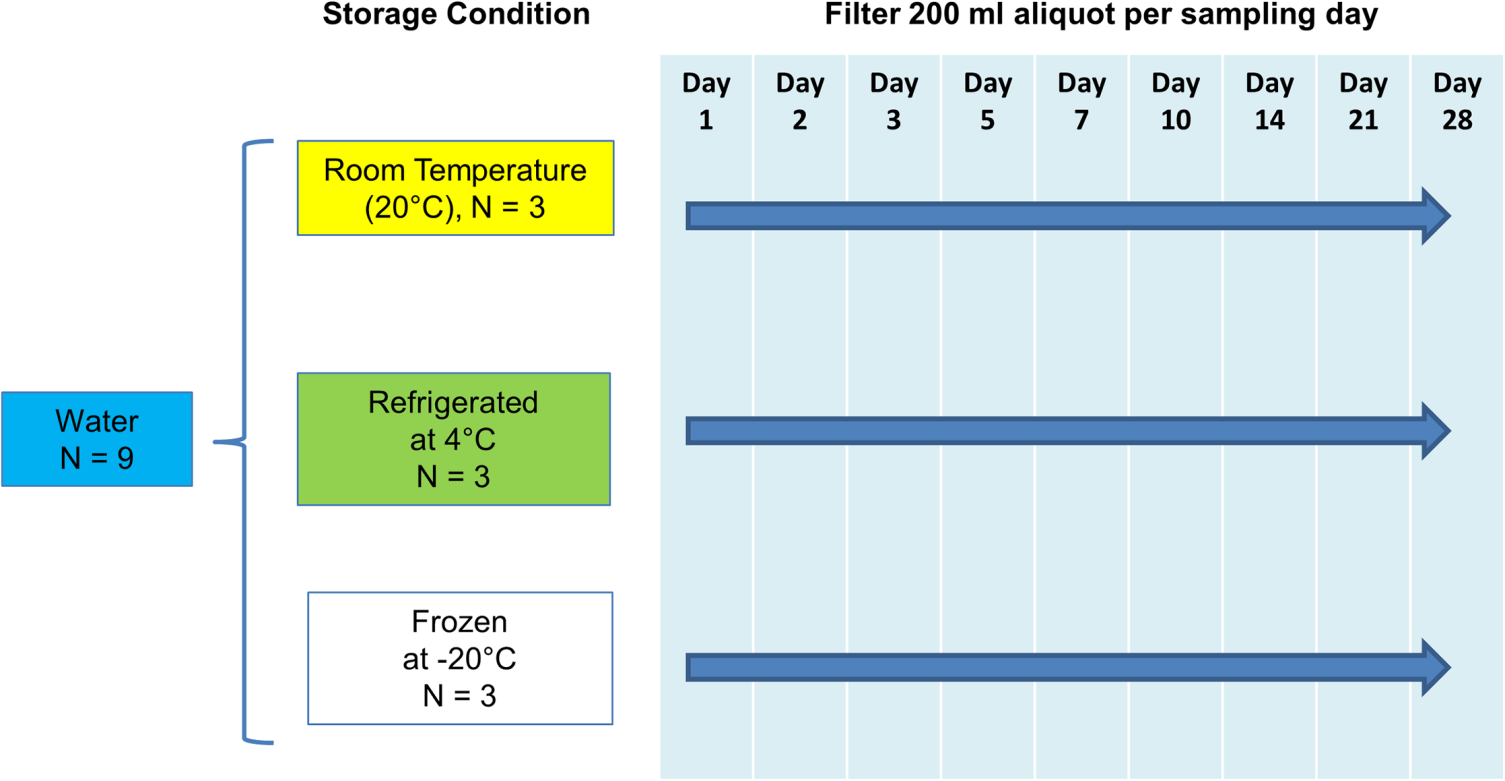
Flow diagram of the experiment investigating the effect of storage method and time on eDNA concentration. *N* refers to number of technical replicates.

We compared the eDNA concentration of water samples stored under three different conditions (room temperature ~20°C, refrigerated at 4°C, frozen at -20°C) over a 28-day period. Twenty litres of water were taken from a 50-L tank containing five adult Oriental weatherloach and placed in a large plastic container with a tap. The container was shaken several times to mix the contents before dispensing into nine 2-L plastic bottles. The bottles were randomly assigned to each of the three storage conditions (three bottles per storage condition treated as technical replicates). A 200ml aliquot from each replicate was filtered through 47mm, 1.2 μm glass fibre filter papers at 1 (within 24h), 2, 3, 5, 7, 10, 14, 21 and 28 days after collection of water samples (Fig 4). DNA from the filter papers was extracted using PowerWater DNA Isolation Kit (Mo Bio Laboratories, Carlsbad, CA) according to the manufacturer’s recommendations.

### Experiment 3B: Effect of storage method and time on eDNA concentration of stream water samples

Stream water was used to validate the findings of Experiment 3A for Day 1 and Day 2. As most eDNA methodologies recommend filtering water for eDNA studies within 24 hours, this experiment investigated the difference in eDNA yield between samples filtered on Day 1 (within 24h of water collection) and Day 2 (after 24h of collection). Twenty litres of stream water was placed inside a 50L plastic tank into which five adult Oriental weatherloach were added. After two days, nine 1-L surface water samples were taken from the tank and randomly distributed among the three storage conditions (room temperature, refrigerated, frozen). Filtration and DNA extraction followed the same procedures as that of Experiment 3A. Filtration of water samples on Day 1 was done within four hours after sample collection and filtration of Day 2 samples occurred 24 hours after the filtration of Day 1 samples.

#### qPCR protocol

DNA extraction and PCR preparation were done in a designated trace DNA laboratory, which is spatially separated from any PCR product. The presence of Oriental weatherloach eDNA for each sample was tested using qPCR primers and probes previously developed for the species [39]. Quantitative PCR reactions were performed in a separate laboratory using the Viia^TM^ 7 Real-Time PCR System (Applied Biosystems^®^, Vic., Australia). PCR reaction mixes consisted of 2μl of DNA template, 1 μl of TaqMan assay, 10 μL of TaqMan Environmental Master Mix (Life Technologies, Carlsbad, CA, USA), 1 μl of Exogenous Internal Positive Control (IPC) Reagent (Applied Biosystems®), 0.2 μL of IPC DNA, and 5.8 μL of PCR water to make a total volume of 20μL. Real-time PCR cycling conditions were set at 50°C (2 min), 95°C (10 min), followed by 55 cycles of 95°C (15 s), 60°C (30 s). Three replicates of synthetic oligonucleotides of the target sequence (details of the synthetic oligonucleotides published in [39]) in a series of 10-fold dilutions with known concentrations ranging from 10^6^ to 10^2^ copies per μL were included in each plate. We quantified the amount of eDNA present in each sample by comparison to these standards. Three PCR replicates were done for each sample. Positive and negative controls, including IPC negatives in three replicates were also included in each run. We considered a reaction positive if an exponential phase was detected during the 55 reaction cycles. We checked for inhibition by looking for delayed or no IPC amplification. A Ct shift of ≥ 3 cycles beyond the blank was regarded as significant inhibition [40].

#### Data analysis

We tested for variation in eDNA recovery across the various treatment groups. The data was assessed for normality of distribution and homogeneity of variances through the Shapiro-Wilk test and Levene’s Test of Equality of Error Variances, respectively [41, 42]. Outliers were determined by inspection of boxplots. Analysis of Variance (ANOVA) was used to test the null hypothesis that all group population means are equal. We used one-way ANOVA for experiments 1 and 2B, two-way ANOVA for experiment 2A and a two-way mixed ANOVA for experiment 3A and 3B. We tested for simple main effects and did post-hoc analyses when a significant interaction was obtained. Epsilon (ε) was calculated according to Greenhouse & Geisser (1959), and was used to correct the mixed ANOVA results when the assumption of sphericity was violated.

The data was log transformed when needed to meet the assumptions of the statistical test. Technical replicates were averaged. When outliers were present, we compared the result of the ANOVA with and without the outliers to see if the outliers substantially affected the result. We kept the outliers in the data set if the results were similar. The significance of all statistical tests was set to α = 0.05. Statistical analyses were conducted using SPSS 21.0 (SPSS Inc., Chicago, USA).

## Results

All positive and negative controls performed as expected. Inhibition was not encountered in any sample. Water sample PCR replicates amplified except for the following: 3 out of 9 PCR wells under the PW-Ethanol treatment in Experiment 1; 2 out of 9 wells under room temperature treatment (Day 28) in Experiment 3A. No inhibition was encountered in all samples. Analyses of synthetic oligonucleotides standards showed that the TaqMan assay performed efficiently: R^2^ ranged from .994 to .999 and PCR efficiency from 86–101%. All data shown below are mean DNA concentration (copies/2μl DNA extract) ± standard deviations, unless otherwise stated.

### Experiment 1: DNA capture, preservation and extraction

**Fig 5:**
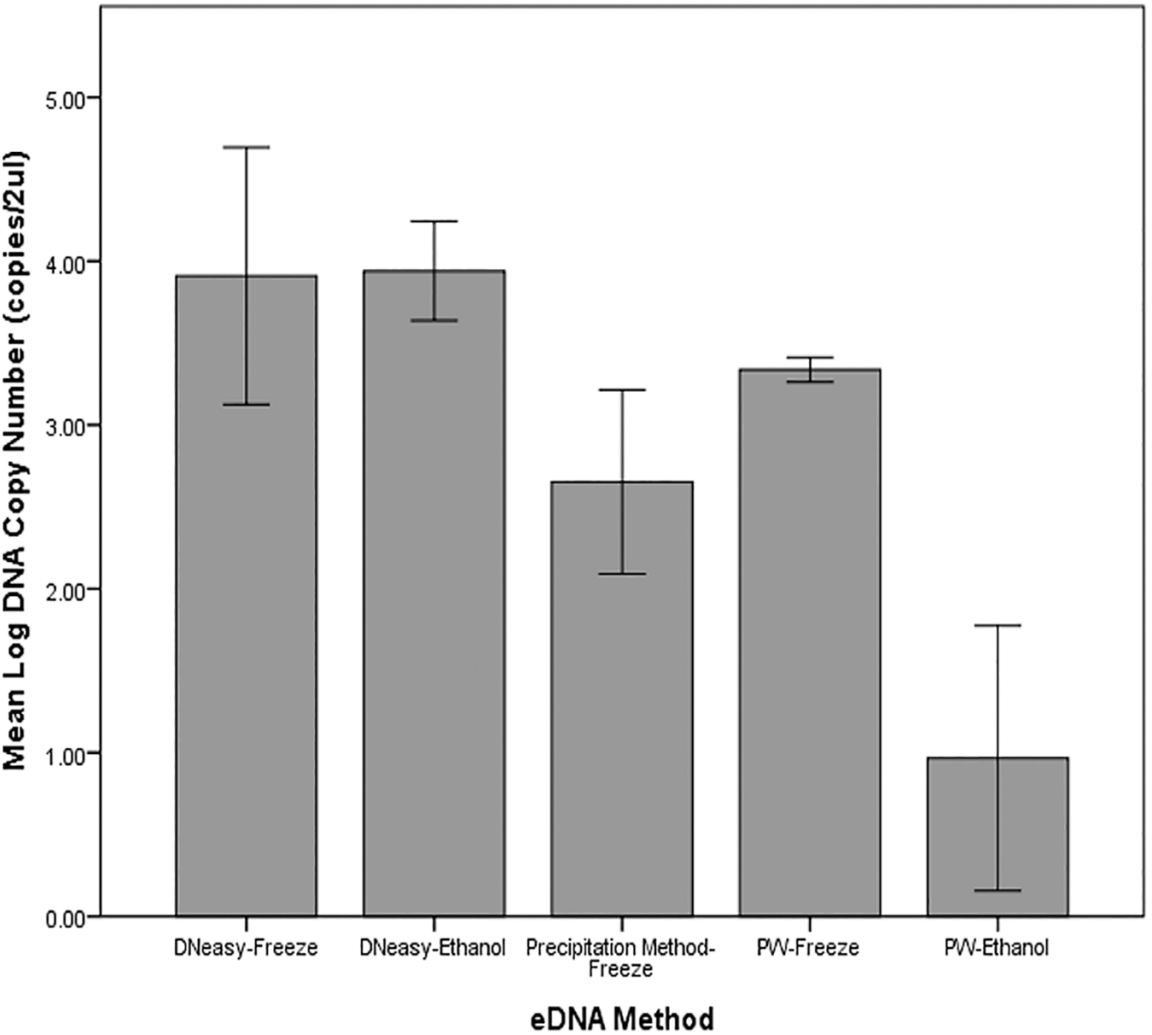
Differences in DNA yield from five different combinations of DNA extraction and storage methods. DNA yield was log_10_ transformed and error bars show the ±2 standard deviation of the mean.

All PCR replicates amplified except for 3 out of 9 PCR wells under the PW-Ethanol treatment. DNA concentrations obtained for experiment 1 produced no outliers and all treatment groups were normally distributed except for one group (PW-Ethanol, p < .0005). Homogeneity of variance as assessed by Levene’s test for equality of variances was violated (p = .007) and thus the data was log-transformed to meet the assumptions of the statistical test. The method which recovered the highest yield (mean DNA copy number in the DNA extract) was the DNeasy-Freeze combination (9629 ± 6563), followed by DNeasy-Ethanol (8920 ± 2276) combination, and the PW-Freeze method (2178 ± 151) (Fig 5). The two methods which recovered the least amount were the Precipitation-Freeze (495 ± 275) and the PW-Ethanol methods (11 ± 9) (Fig 5). DNA copy number was significantly different for the different eDNA methods, *F* (4, 10) = 83.467, *p* < .0005, ῶ^2^ = 0.956. Tukey’s post-hoc test revealed no significant difference between the eDNA yield of the top three performing methods (p < .05) but significant differences in yield was observed in all comparisons with the Precipitation-Freeze method and PW-Ethanol (S2 Table).

There was a variation in the volume of water sample processed for the Precipitation-Freeze method (15ml) compared to the other methods (250ml). Table 1 shows the results as DNA copies per unit water volume, in addition to copies per DNA extract. When presented as number of DNA copies per ml of water sample processed, the Precipitation-Freeze method gave a mean DNA yield comparable to the yield of the DNeasy-Freeze and DNeasy-Ethanol methods (Table 1).

**Table 1:** Results of Experiment 1 presented as number of copies per unit DNA extract and number of copies per unit water volume. The volume of processed water samples, DNA extraction kits and final DNA elution volumes are also given.

	Volume of water sample processed (ml)	DNA extraction kit	DNA elution volume (μl)	DNA concentration from qPCR (DNA copies in 2 μl DNA extract)	Equivalent number of DNA copies in entire DNA extract (or entire water sample)	Equivalent number of DNA copies per ml of water sample
**DNeasy-Freeze**						
Sample 1	250	DNeasy	200	3921	392100	1568
Sample 2	250	DNeasy	200	8166	816600	3266
Sample 3	250	DNeasy	200	16800	1680000	6720
Mean				9629	962900	3852
**DNeasy-Ethanol**						
Sample 1	250	DNeasy	200	6303	630300	2521
Sample 2	250	DNeasy	200	10020	1002000	4008
Sample 3	250	DNeasy	200	10436	1043600	4174
Mean				8920	891967	3568
**Precipitation-Freeze**						
Sample 1	15	DNeasy	200	299	29900	1993
Sample 2	15	DNeasy	200	809	80900	5393
Sample 3	15	DNeasy	200	376	37600	2507
Mean				495	49467	3298
**PowerWater-Freeze**						
Sample 1	250	PowerWater	100	2036	101800	407
Sample 2	250	PowerWater	100	2336	116800	467
Sample 3	250	PowerWater	100	2161	108050	432
Mean				2178	108883	436
**PowerWater-Ethanol**						
Sample 1	250	PowerWater	100	22	1100	4
Sample 2	250	PowerWater	100	6	300	1
Sample 3	250	PowerWater	100	6	300	1
Mean				11	567	2

### Experiment 2: Comparison of different combinations of filter paper and DNA extraction method

#### A. Samples from tap water

**Fig 6:**
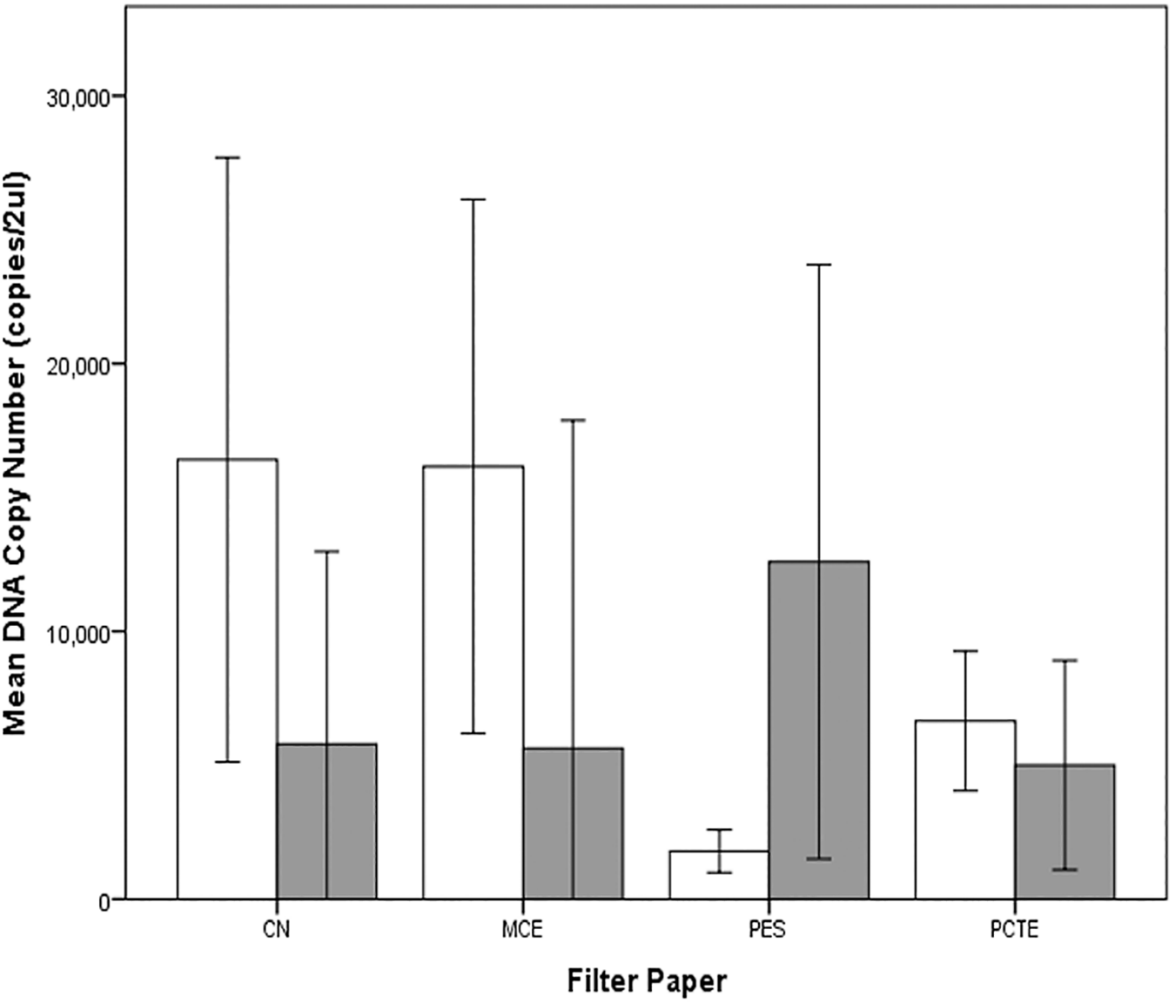
DNA yield from eight DNA extraction kit-filter paper combinations from samples in aquaria with UV-sterilized water. Clear bars used DNeasy extraction kit while shaded grey bars used the PowerWater kit. CN = Cellulose Nitrate, MCE = Mixed Cellulose Ester, PES = Polyethersulfone, PCTE = Polycarbonate track-etched. Error bars show the ±2 standard deviation of the mean.

The thickness of the 0.8μm GF filter papers caused leakage of water samples during filtration, thus, data for the GF filter papers were not included in the analysis. The data exhibited homogeneity of variances (*p* = .088) and all DNA extraction kit-filter paper groups exhibited normal distributions except for PW-MCE (p = .010). We kept outliers in the data set since running the ANOVA with and without outliers gave similar results. There was a significant interaction effect between filter paper and extraction kit on DNA copy numbers, *F* (3, 32) = 14.265, p < .0005, partial ƞ^2^ = .572. Calculation of simple main effects for DNA extraction kit revealed that extraction kit had a significant effect on DNA yield when paired with the filter papers CN, MCE and PES. For CN, mean DNA yield for samples extracted with DNeasy was 16409 ±5643 and 5796 ±3953 for samples extracted with the Power Water kit, a statistically significant mean difference of 10,613 (95% CI, 5157 to 16069), *F* (1,32) = 15.697, *p* < .0005, partial ƞ^2^ = .329. For MCE, DNeasy-extracted samples had a mean yield of 16160 ±4983 copies compared to Power Water-extracted samples with mean yield of 5636 ± 6122. This was a significant mean difference of 10524 (95% CI, 5068 to 15980), *F* (1, 32) = 15.435, p < .0005, partial ƞ^2^ = .325. For PES, DNA yield was higher in samples extracted with the Power Water kit (12598 ± 5548), a significant mean difference of 10809 (95% CI, 5352 to 16265), *F* (1, 32) = 16.281, *p* < .0005, partial ƞ^2^ = .337 (Fig 6, S3 Table).

Simple main effects for filter paper showed that filter paper type had a significant effect on DNA yield when extracted with either DNeasy (*F*(3,32) = 14.623, p < .0005, partial ƞ^2^ = .578) or Power Water (*F*(3,32) = 3.566, *p* = .025, partial ƞ^2^ = .251). When DNeasy extraction kit was used, samples filtered on CN and MCE yielded significantly higher DNA copies compared to PES and PCTE. The differences in mean yield between CN and MCE and between PES and PCTE were not significant. For the Power Water Kit, a significant mean difference in DNA yield was only seen between PES and PCTE, with a higher mean copy number extracted on PES samples compared to PCTE. Pairwise comparisons for the simple main effect for filter paper including 95% confidence intervals and statistical significance for all DNA extraction kit-filter paper combinations are in S4 Table.

#### B. Samples from stream water

**Fig 7:**
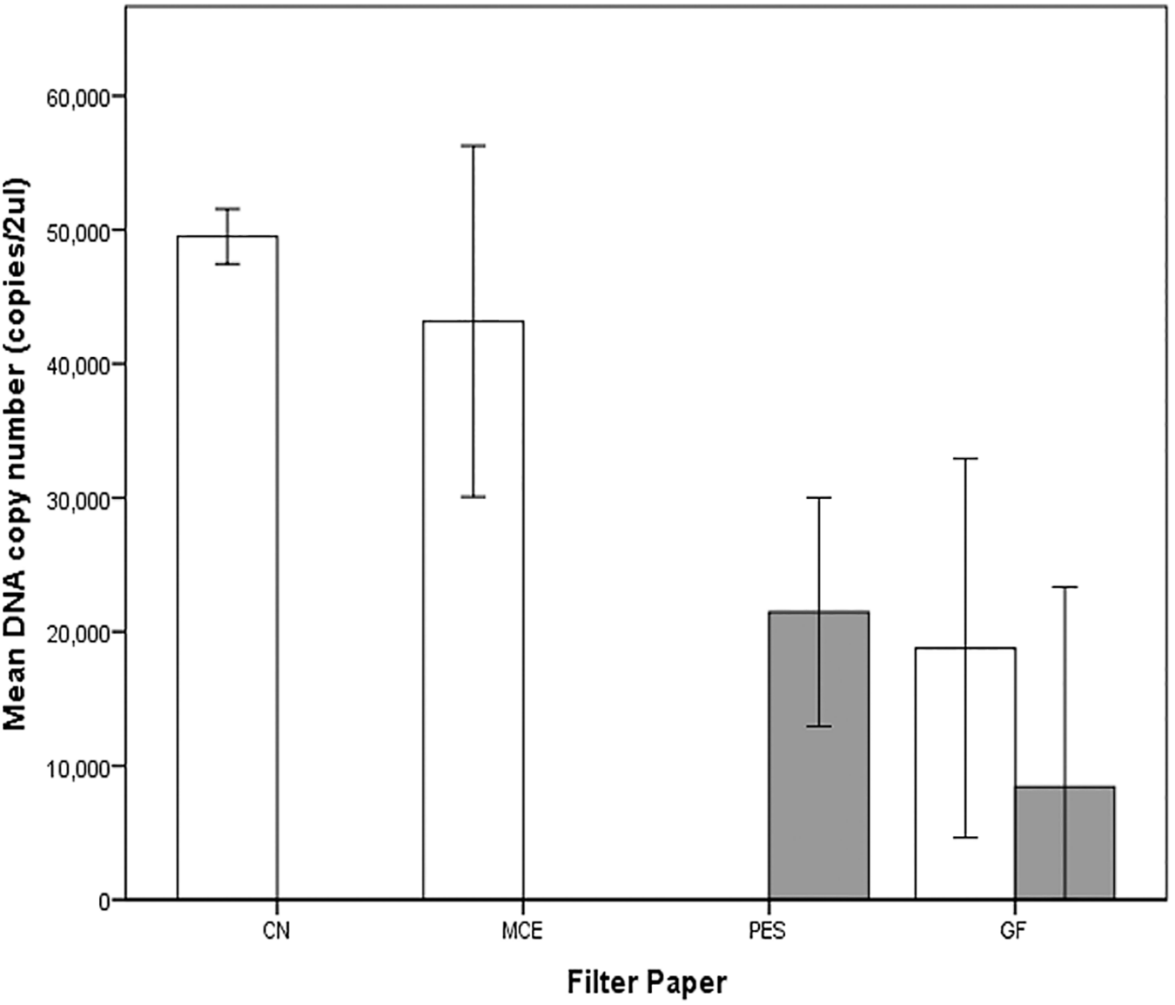
DNA yield from five different extraction kit-filter paper combinations from stream water samples. Clear bars used DNeasy extraction kit while shaded grey bars used the PowerWater kit. Error bars show the ±2 standard deviation of the mean.

The data exhibited homogeneity of variance (*p* = .314) and normality except for one extraction kit-filter paper combination (PW-PES, p = .006). No outliers were observed. DNA yield was significantly different among the DNA extraction kit-filter paper combinations, *F* (4, 10) = 26.872, *p* < .0005, ῶ^2^ = 0.873. The combinations which gave the highest DNA yields were: DNeasy-CN (49497 ±1027), DNeasy-MCE (43160 ± 6550), and PW-PES (21472 ± 4264). DNeasy-GF (18777 ± 8073) and PW-GF (8433 ± 7455) had the lowest DNA yield (Fig 7). Tukey post hoc analysis revealed significant differences in mean DNA yield when DNeasy-CN was compared with PW-PES (*p* = .001), DNeasy-GF (*p* = .001) and PW-GF (*p* < .001). Significant differences in mean DNA yield were also seen when DNeasy-MCE was compared with PW-PES (*p* = .007), DNeasy-GF (*p* = .003) and PW-GF (*p* < .001). Differences in mean DNA yield did not differ significantly when PW-PES, PW-GF and DNeasy-GF were compared to each other (Fig 7).

We found significant differences in the time it took to filter 500ml of samples through the different filter paper types, Welch’s *F*(3, 3.564) = 41.632, *p* = .003. The filter paper with the fastest filtration time is GF (0.8 ± .1 min), followed by CN (1.6 ± 0.4 min) and PES (18.2 ± 3 min). Filtration through MCE took the longest time at 175 ± 38 min. Table 2 gives a summary of DNA yield, filtration time, flow rates and cost per sample for Experiment 2B.

**Table 2:** Performance and cost of filter paper-DNA extraction kit combinations in Experiment 2B. CN = Cellulose Nitrate, MCE = Mixed Cellulose Ester, PES = Polyethersulfone, GF = Glass Fibre filter paper.

Filter Paper	DNA Extraction Kit	eDNA yield	Flow rate	Cost per filter paper (US$)	Cost per DNA extraction (US$)	Combined cost of filter paper & extraction (US$)
CN	DNeasy	High	Moderate	0.44	4.5	4.9
MCE	DNeasy	High	Very Slow	2.0	4.5	6.5
PES	PowerWater	Medium	Slow	1.9	8.5	10.4
GF	DNeasy	Medium	Fast	0.25	4.5	4.7
GF	PowerWater	Medium	Fast	0.25	8.5	8.7

### Experiment 3A. Effect of storage method and time before filtration on DNA yield

#### A. Samples from UV-sterilized tap water

**Fig 8:**
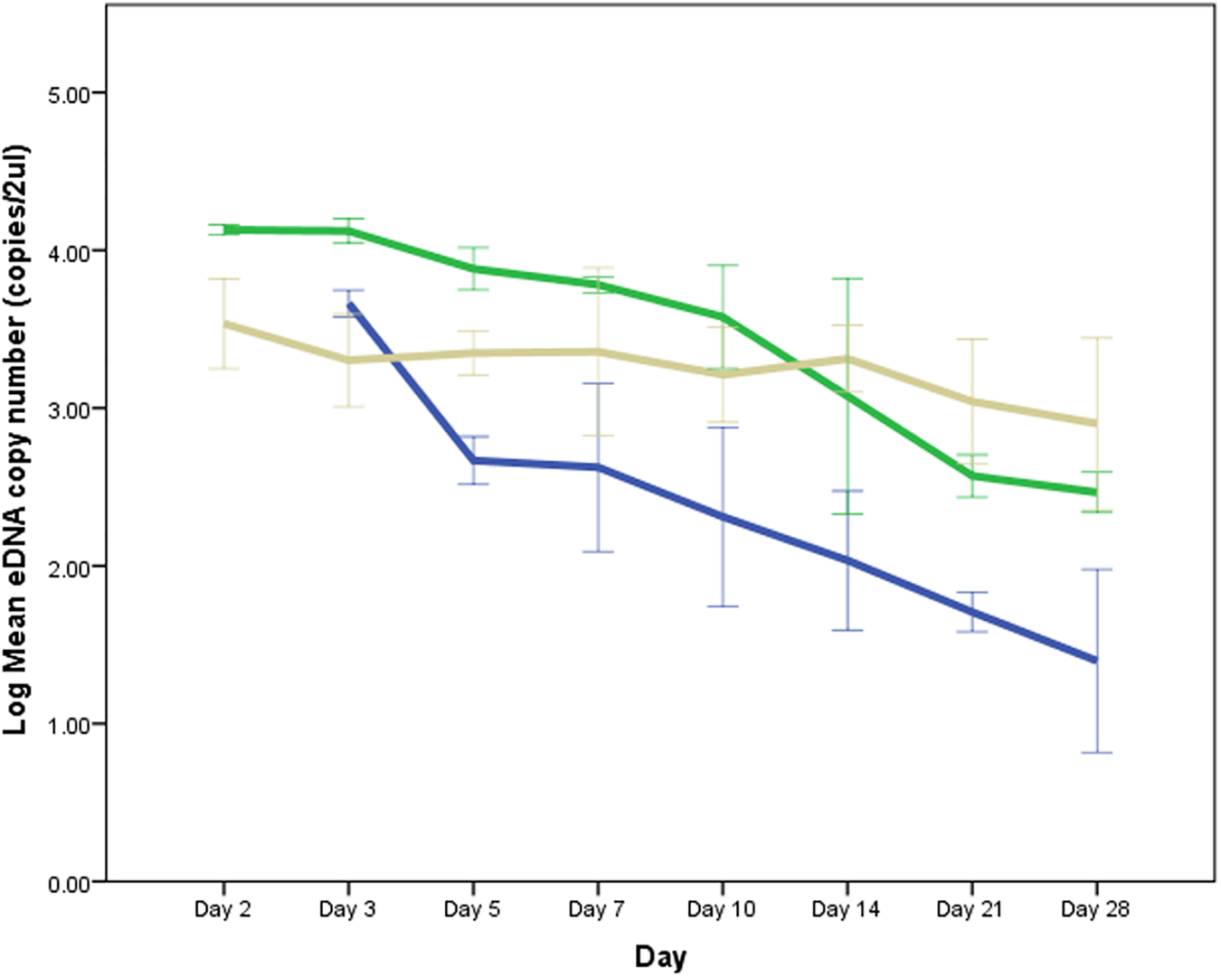
Changes in DNA yield across time for tap water samples stored under three different conditions: Room temperature (blue line), refrigerated (green line) and frozen (brown line). Error bars show the ±2 standard deviation of the mean.

Data for Day 1 (all storage conditions) and Day 2 (room temperature) were excluded from analysis due to a DNA extraction error which affected downstream applications. The data for the rest of the samples under the three storage conditions exhibited homogeneity of variances and normal distributions after log transformation, except for two groups (Day 10- refrigerated and Day 28-room temperature) which still exhibited non-normality after transformation. The assumption of sphericity was violated for the two-way interaction, *x*^*2*^(20) = 44.879, *p* = .005, thus, a Greenhouse-Geisser correction was used. There was a significant interaction between storage method and time, *F* (4.430, 13.291) = 10.117, *p* < .001, partial ɳ^2^ = .771. Simple main effects for storage method revealed a significant difference in copy numbers between methods across time points in the experiment. DNA copy numbers for all storage methods were significantly different from each other on days 2, 3, 5 and 21 (*p* < .001) (Fig 8). For days 7, 10, 14 and 28, significant differences in DNA yield were seen only when room temperature samples were compared with refrigerated and frozen samples (S5 Table).

Testing for the simple main effect for time showed a significant effect of time on copy number for samples stored under room temperature *F*(1.239, 2.478) = 36.436, p = .015, partial ɳ^2^ = .948, and refrigeration *F*(1.277, 2.554) = 53.088, *p* = .009, partial ɳ^2^ = .964, but not for frozen samples. For room temperature samples, a significant reduction in copy numbers were seen between day 3 and all subsequent time points, day 5 and days 21 and 28, and day 7 and days 14 and 21. For refrigerated samples, significant differences were seen between day 2 and days 5, 7, 10, 14, 21 and 28, between day 3 and the rest of the time points, day 5 and days 14, 21 and 28, day 7 and days 21 and 28, and finally day 10 and days 21 and 28. P-values of significant pairwise combinations for the simple main effects of time are in S6 Table.

#### B. Samples from stream water (Day 1 and Day 2)

**Fig 9:**
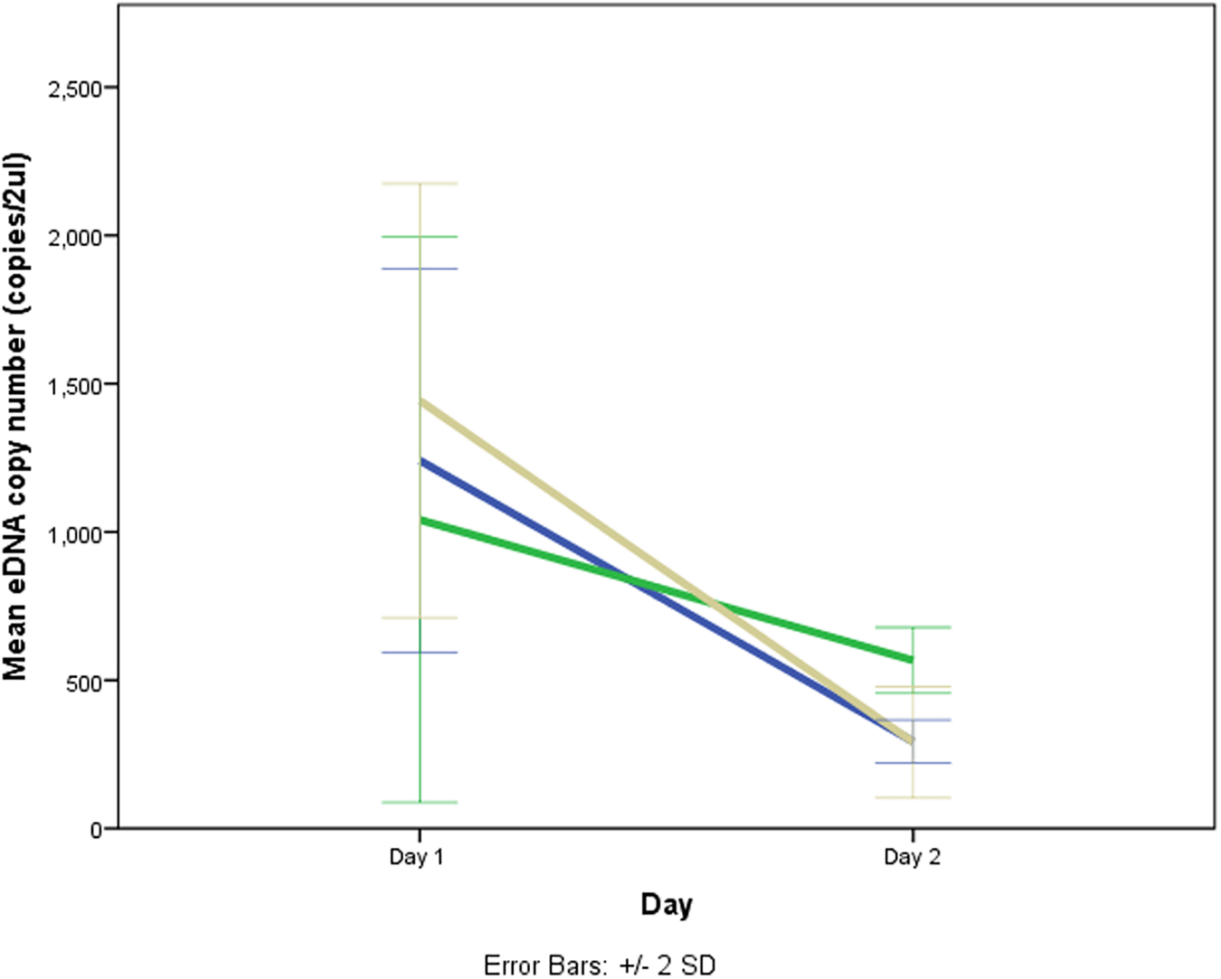
Changes in DNA yield from Day 1 to Day 2 for stream water samples stored under three different conditions. Blue line represents room temperature (20°C), green line represents refrigerated (4°C) and brown line represents frozen (-20°C). Error bars show the ±2 standard deviation of the mean.

The data met all the assumptions for the mixed ANOVA. There was no significant interaction between storage method and time on DNA yield for Day 1 and 2, *F* (2,6) = 1.831, *p* = .237, partial ɳ^2^ = .379. The main effect of time showed a significant difference in mean DNA copy number between day 1 and day 2 for all the storage methods, *F*(2,6) = 2.54, *p* = .784, partial ɳ^2^ = .848 (Fig 9). The main effect of storage methods showed that there was no significant difference in mean DNA copy number between different storage methods, *F*(2,6) = .254, *p* = .784, partial ɳ^2^ = .078.

## Discussion

Our laboratory experiments revealed that choice of capture, preservation, filtration and extraction methods can significantly affect DNA yield. In addition, we demonstrated how storage method and time prior to filtration affected the number of target DNA copies retrieved. PCR detection of eDNA relies on the number of copies in the DNA extract, thus, maximizing yield is the best way to increase detection.

We found that the filtration method, combined with either ethanol or freezer storage, and extracted with either Qiagen’s DNeasy or MoBio’s PowerWater kit (except samples stored in ethanol and extracted with PowerWater) yielded better results compared to the precipitation method, which is consistent with previous studies [19, 21]. It is highly probable that the reason we obtained this result is because we filtered 250 ml of water compared to just 15ml for the precipitation and centrifugation method, which is likely to contribute a disproportionate amount of eDNA between samples. However, a previous study that compared filtration and the precipitation method using 15ml water samples still found that filtration recovered more DNA than the precipitation method [18]. In Table 1, we adjusted the eDNA concentration hypothetically should the entire 250-ml undergo the precipitation method and the results were comparable to those obtained by filtration. Nevertheless, the precipitation method could only process a limited volume at one time, and processing the same amount of water as that of other methods to get the same amount of DNA would be impractical. Thus our results support the use of the filtration method when larger volumes of water samples for eDNA analysis are available. We also found that eDNA yield was higher for samples extracted with the DNeasy kit compared to the PowerWater kit, although this was not statistically significant in our study. Previous studies, however, found that Qiagen DNeasy kits extracted a significantly greater amount of eDNA compared to the PowerWater kit [19, 43]. We used manufacturers’ recommendations in eluting DNA in this study (200 μl buffer for DNeasy samples, 100 μl for Powerwater), thus the difference in elution volumes could have led to the non-significant difference in DNA yield between the two extraction kits. Nevertheless, the information that DNA yield was still higher with the DNeasy kit despite a higher elution volume is useful, particularly for researchers who would like to get the maximum amount of DNA extract without sacrificing DNA concentration. In applied situations where attempts are made to detect species at extremely low-densities, variability in recovery of just a few target molecules may signify the difference between failed or successful detection. In this instance, the higher DNA yield in DNeasy-extracted samples could play an important role in eDNA’s application to inform on-ground management.

Our results reveal that the preservation of filter papers using 95% ethanol and extracted with the Qiagen DNeasy kit is comparable to frozen storage. Filters stored in ethanol and extracted with the PowerWater kit recovered significantly less eDNA suggesting that ethanol-stored samples are not compatible with the PowerWater kit. The storage of filters in ethanol is advantageous in field situations where access to ice is restricted or when samples cannot be frozen immediately. Other ambient temperature buffers, such as cetyl trimethyl ammonium bromide (CTAB) and Longmire’s solution, have also been successfully used to preserve eDNA [20, 44]. Compared to these buffers however, ethanol is widely available, inexpensive and can be used straightaway whereas the other solutions have to be prepared using several ingredients. A recent eDNA study [45] also found no significant difference in eDNA copies from filter papers stored for up to six days in ethanol. Our study suggests that ethanol preservation can be a practical alternative when other buffers are not available. Studies looking at long-term preservation of filter papers in ethanol and other ambient temperature buffers for eDNA studies are recommended.

A previous study found no significant difference in DNA copy numbers between CN and PES filter papers of the same pore size (0.8μm) [20]. In contrast, we found statistically significant differences in DNA yield between CN and PES extracted with the DNeasy kit in our study. The difference in the results between the two studies is likely due to the different combinations of DNA storage/preservation and extraction methods used. Renshaw, Olds [20] stored samples in CTAB and used the PCI extraction protocol, whereas we stored the filter papers in a -20°C freezer prior to DNA extraction using the DNeasy kit. As the results of this study reveal, there is a significant interaction effect between the type of filter paper and the extraction method on DNA copy numbers. The storage/preservation method also affects DNA yield. It is clear from our study that different combinations of storage, extraction, and choice of filter paper can have a significant impact on DNA yield.

Our results agree with the findings of Liang and Keeley [37] who examined the effect of filter paper type on the recovery of spiked DNA plasmid. They found that DNA had different binding affinities to different types of filter paper. MCE recovered the most DNA, followed by Polyvinylidene Fluoride (PVDF—not trialled in the present study), PES and polycarbonate filter papers [37]. Liang and Keeley [37] used the PowerSoil® DNA Isolation Kit to extract DNA from the filter papers. In our study, we also found that for DNeasy-extracted samples, MCE gave a significantly higher DNA yield compared to PES and Polycarbonate filters. However, if PowerWater is used for DNA extraction, a significant difference in DNA yield was only seen between PES and PCTE. It is worthwhile to note that although our results agree with the findings of Liang and Keeley [37], the main difference between the studies was that they used purified DNA while our study dealt with eDNA which consists of both extra- and intracellular DNA, and even small tissue fragments or feces. Thus, while we could not calculate the efficiency of the methods compared, our experimental methodology enabled us to potentially capture eDNA in all its forms from the water samples.

The different binding affinities of DNA to various filter material could in part be explained by the inherent properties of the filter itself. For instance, filter papers can be categorized as depth filters (particles retained on the surface and within the filter matrix, i.e. GF, CN, MCE) or surface filters (particles trapped on filter’s surface, i.e. PES, PCTE) (GE Healthcare, 2013). A possible explanation why CN and MCE yielded significantly more DNA than the other filter papers (for DNeasy-extracted samples only) could be because DNA was also trapped within the matrix itself and not only on the surface. Another explanation could be the inherent high DNA and protein binding capacity of cellulose nitrate filters [46, 47]. Further investigation is required to determine the factors that allow eDNA to bind to some filter types more than others. Testing the performance of other filter papers not included in this study (e.g. nylon, PVDF) is also recommended.

Differences in the extraction methodology could explain the higher DNA yield we saw when CN and MCE were extracted with the DNeasy kit compared to the PowerWater kit. For instance, the DNeasy extraction process we used involved one hour incubation in tissue lysis buffer and Proteinase K. This step could have resulted in the release of more nucleic acids from within the filter matrix. The DNeasy method relies on a biochemical method to lyse cells compared to the PowerWater method which uses a mechanical method (bead beating) [18]. The PowerWater extraction process also involved several transfers of the supernatant to different collection tubes. Gaillard and Strauss [48] found that significant amounts of DNA can stick to the walls of polypropylene tubes resulting in loss of DNA. This could have contributed to the lower yield in the PowerWater extracts.

It is of interest that the DNeasy-GF and PowerWater-GF combinations gave the lowest DNA yield particularly because these are two of the most widely used extraction-filter paper combinations in eDNA studies (e.g. [3, 9, 29, 39, 49–52]). Several studies have found that the PCI or the CTAB method yield more eDNA compared to commercial DNA extraction kits [18, 20, 53]. The DNA kits are convenient and simple to use but are more expensive. In contrast, the PCI and CTAB methods are inexpensive but require careful preparation and handling of toxic chemicals. This study, along with several others, provides guidance for researchers to choose their method depending on research objectives, personal preference, ease of use and availability of resources.

Flow rate is also important when choosing filter papers for eDNA studies. Filter papers with higher flow rates can significantly decrease filtration time (potentially contributing to eDNA degradation during long filtration) and associated labour costs. Filtration time can be a crucial factor when using highly turbid samples. For example, we have experienced filtration times of up to 3 hours for 2L samples from highly turbid waterways (>200 nephelometric turbidity unit (NTU)), despite using multiple 1.2 μm glass fibre filter papers. Previous work by Robson, Noble [35] suggested increasing filter pore size (10 or 20 μm) or using pre-filters to decrease filtration time particularly when using water with high sediment load or algae. This could mean though that higher water volumes must be filtered in order to get the same quantity of eDNA if one is to use smaller pore sized filters. Turner, Barnes [54] found that using 0.2μm filter paper maximized DNA capture but could only filter very small volumes due to clogging. They recommended calculating pore size and water volume isoclines to attain identical amount of carp DNA. Our study shows that choosing filter papers with higher flow rates can also decrease filtration time.

Aside from DNA yield and flow rate, cost of filter papers is another factor to consider in filter paper choice. The filter paper type can significantly increase eDNA processing costs, not only because of the price of the filter paper but also because filters with low flow rates increases filtration time and labour costs. Our study suggests cellulose nitrate filter papers extracted with Qiagen’s DNeasy kit as the most cost-efficient combination. We recognize however that eDNA methods vary depending on the objectives of the study. If large quantities of water samples are to be filtered, then perhaps using glass fibre filters or CN filter paper of larger pore size would be more appropriate. For limited sample volumes, the CN-DNeasy combination could be more cost-efficient.

A common practice for many eDNA studies is to filter water samples within 24 hours. Our results support this practice as we have observed a significant decrease in DNA copy numbers from Day 1 to Day 2 in stream samples regardless of storage method. If samples cannot be filtered within 24 hours, our results suggest that refrigeration at 4°C for a few days would yield higher DNA copies compared to freezing. For long-term storage, water samples should be placed inside a -20°C freezer. While this study looked at the effect of storage temperature on eDNA, it is also important to note that eDNA degradation is affected by a range of other physiochemical factors such as light, pH, conductivity and enzymatic activity [34, 37]. These factors can interact in a synergistic or antagonistic manner in relation to DNA degradation [37]. Thus, while our experiment produced such results, it is also possible that the superiority of the storage conditions (room temperature, refrigerated, frozen) could vary depending on the characteristics of the water samples.

Takahara, Minamoto [31] investigated the effect of freezing and thawing water samples on eDNA detection and concentration of common carp (*Cyprinus carpio)* DNA. They found that detection of common carp DNA was lower in samples that underwent freezing (frozen for 2–5 days) and thawing compared to samples that did not, although no significant difference in DNA concentration was seen. In our experiment, we found no difference in detection rates but found significantly more DNA copy numbers on Day 1 aliquots (unfrozen, filtered within 4 hours) compared to Day 2 aliquots (stored in freezer for 1 day) from stream water samples. For our tap water aquarium samples, we measured eDNA yield after several freezing and thawing events and showed that the repeated freezing and thawing process from Day 2 to Day 28 did not significantly affect the DNA copy number, suggesting that the first freeze-thaw cycle is the crucial factor which significantly affects eDNA concentration. Some studies have investigated the effect of the freeze-thaw cycle on DNA in intact cells (such as spermatozoa) and have found that the freezing–thawing process affects DNA integrity by causing strand breaks [55, 56]. Environmental DNA analysis however already deals with short DNA sequences and it seems that subsequent freeze-thaw cycles after the first one do not significantly affect the DNA concentration of these short fragments.

Our experiments were set-up in the laboratory and thus represented ideal conditions for eDNA analysis. Although we were able to validate our filter paper-DNA extraction kit experiment with stream water samples, we were only able to test Day 1 and Day 2 for the storage method and time experiment. We recommend exploring the same experimental set-up using actual field samples for the entire duration of the experiment to see if similar results will be obtained.

There are also many other possible combinations of eDNA methods that we have not tested here. In particular, this study was not able to include other commonly-used extraction and preservation methods used in eDNA studies such as PCI extraction and preservation with Longmire’s solution. We also acknowledge that our methods comparison study investigates variation in DNA yield only up to the DNA extraction stage and that subsequent variation in PCR reagents, assay design and set-up procedures could also affect DNA quantity and detection [36, 57]. For instance, we did not encounter inhibition in our samples when using any of the two DNA extraction kits; however, Eichmiller, Miller [19] observed more inhibition in DNeasy-extracted samples compared to Power Water-extracted samples in a previous study. It may be that our samples were free of inhibitors or, alternatively, the use of the TaqMan® Environmental Master Mix in the PCR master mixes in this study have reduced any effect of inhibitors as it has been reported that this reagent can effectively release inhibition in eDNA samples [57, 58].

## Conclusion

The choice of eDNA methods should consider efficiency, reliability and comparability in addition to cost and ease of use. In this study, we have provided further evidence that the choice of eDNA capture, storage, extraction method and filtration materials can substantially affect DNA yield. By using similar pore-sized filters, we were able to directly compare eDNA yield from different filter papers using two extraction kits. We recommend filtration with cellulose nitrate filter paper and extraction with the Qiagen’s DNeasy kit for commercial DNA kit users. Filters can either be stored frozen before extraction or placed in ethanol for up to four days without significantly affecting DNA copy numbers. Our results support the recommendation to filter water samples within 24hours but if this cannot be done, our results suggest short-term refrigeration for up to five days may be a better storage option than freezing. The information provided in this study has practical implications for eDNA field studies and is useful in designing eDNA studies while considering resource costs and available resources. The result of this study is likely to be of particular importance to eDNA detection of low-density invasive or threatened species, where small variations in DNA recovery can signify the difference between detection success or failure.

## Supporting information

S1 Appendix DNA copy numbers from qPCRs for all experiments. (XLSX)

S1 Table Product details of filter papers used in the experiments. (XLSX)

S2 Table Multiple Pairwise comparisons of eDNA methods using Tukey’s HSD for Experiment 1: DNA capture, preservation and extraction. (XLSX)

S3 Table Multiple Pairwise comparisons of the simple main effects for DNA Extraction kit for Experiment 2A. (XLSX)

S4 Table Multiple Pairwise comparisons of the simple main effects of filter paper for Experiment 2A. (XLSX)

S5 Table Multiple Pairwise comparisons of the simple main effects of time for Experiment 3A. (XLSX)

S6 Table Multiple Pairwise comparisons of the simple main effects of storage method for Experiment 3A. (XLSX)
